# Integrated analysis identifies an aging-related prognostic signature for lung adenocarcinoma

**DOI:** 10.1186/s12885-026-16534-w

**Published:** 2026-08-07

**Authors:** Rui Zhang, Xinyi Chen, Na Li, Min Peng

**Affiliations:** Cancer Center, Renmin Hospital of Wuhan University, Wuhan, 430060 China

**Keywords:** Lung adenocarcinoma, Aging, Prognosis, Immune microenvironment, CLDN14

## Abstract

**Background:**

Lung adenocarcinoma (LUAD) is characterized by marked molecular heterogeneity, resulting in substantial variability in clinical outcomes. Aging is a major risk factor for LUAD and is closely associated with genomic instability, metabolic dysregulation, cellular senescence, and remodeling of the tumor microenvironment. However, the prognostic significance and biological relevance of aging-related genes in LUAD remain incompletely understood. This study aimed to systematically characterize aging-related molecular alterations in LUAD and develop a prognostic signature with potential biological and clinical relevance.

**Methods:**

Transcriptomic and clinical data for LUAD were obtained from The Cancer Genome Atlas (TCGA), and aging-related genes were collected from a public aging-associated gene set. Differentially expressed genes (DEGs) were identified by comparing tumor and normal tissues and intersected with genes from the GEO validation cohorts GSE37745 and GSE50081 to obtain common aging-related DEGs. Prognosis-associated genes were screened by univariate Cox regression, and molecular subtypes were identified by consensus clustering. An aging-related prognostic signature was then constructed using LASSO-Cox regression and validated in two independent GEO cohorts. Associations of the risk score with clinicopathological features, somatic mutation patterns, stemness, and the tumor immune microenvironment were further explored. In addition, single-cell RNA-seq data from GSE131907 were used to investigate the cellular distribution of a key model gene, while RT-qPCR analysis of clinical samples and in vitro experiments were performed for preliminary validation.

**Results:**

A total of 274 aging-related DEGs were identified in LUAD, of which 52 were significantly associated with overall survival. Based on these genes, LUAD samples were classified into two molecular subtypes with distinct prognoses, immune infiltration patterns, and biological pathway enrichment characteristics. An eight-gene prognostic signature consisting of CD19, CYP17A1, TFAP2A, HMGA2, ABCC2, PCSK9, CLDN14, and INHA was subsequently established. This signature stratified patients into high-risk and low-risk groups with significantly different overall survival in the TCGA cohort, and its prognostic relevance was further evaluated in the GSE37745 and GSE50081 cohorts. The risk score was independently associated with prognosis and correlated with advanced clinicopathological features. High-risk tumors exhibited higher stemness, a higher somatic mutation frequency, and enrichment of proliferation-related pathways, whereas low-risk tumors displayed stronger immune-related characteristics and higher immune and stromal scores. Single-cell analysis showed that CLDN14 had relatively higher expression in B cells than in other annotated cell populations. RT-qPCR analysis in paired tumor and adjacent normal tissues from 10 LUAD patients further showed that CLDN14 was significantly upregulated in tumor tissues. In vitro assays suggested that CLDN14 knockdown impaired the proliferative, migratory, and invasive capacities of LUAD cell models.

**Conclusions:**

We established and validated an aging-related eight-gene prognostic signature for LUAD with independent prognostic value. This signature was associated with distinct clinical, molecular, stemness, mutational, and immune microenvironmental features. In addition, CLDN14 was identified as a candidate gene potentially associated with LUAD progression and was supported by preliminary experimental validation. These findings provide an initial aging-related framework for prognostic stratification and biological characterization in LUAD.

**Supplementary Information:**

The online version contains supplementary material available at https://doi.org/10.1186/s12885-026-16534-w.

## Introduction

Lung adenocarcinoma (LUAD), the most common histological subtype of non-small cell lung cancer, remains a leading cause of cancer-related mortality worldwide [1, 2]. Despite substantial advances in surgery, chemotherapy, targeted therapy, and immune checkpoint blockade, patient outcomes remain highly heterogeneous [3, 4]. Even among patients with similar clinicopathological characteristics who receive comparable treatments, survival can vary markedly, reflecting the pronounced molecular and biological heterogeneity of LUAD [5]. Recent studies have further shown that LUAD progression and treatment failure are driven by diverse molecular mechanisms, including transmembrane mucin-mediated tumor progression and immune microenvironment remodeling, circRNA-related cell-cycle regulation, m6A-associated metabolic reprogramming, DNA repair-mediated chemoresistance, and signaling pathways involved in osimertinib resistance [6–10]. These challenges highlight the need for robust biomarkers and prognostic models that can better capture disease heterogeneity and facilitate individualized management.

Aging is a major risk factor for LUAD, with both incidence and mortality increasing with advancing age [11, 12]. Beyond chronological age, aging is accompanied by interconnected molecular alterations, including genomic instability, epigenetic dysregulation, mitochondrial dysfunction, chronic inflammation, and cellular senescence, all of which may contribute to tumor initiation and progression [13]. Among these hallmarks, cellular senescence has emerged as an important and context-dependent process in cancer biology [14, 15]. Although senescence was traditionally viewed as a stable state of cell-cycle arrest that suppresses malignant transformation, senescent cells can also acquire a senescence-associated secretory phenotype (SASP). Through the release of cytokines, chemokines, growth factors, and matrix-remodeling factors, SASP can reshape the tumor microenvironment and influence inflammation, immune remodeling, stemness, metastasis, and therapeutic response [16–19]. Therefore, aging-related genes may reflect biologically meaningful tumor states and serve as informative biomarkers for prognostic assessment in LUAD [20].

Although several aging- or senescence-related prognostic signatures have been reported for LUAD, most have been developed primarily from bulk RNA-sequencing data and remain limited in biological interpretability and translational relevance [21–23]. Existing studies have provided valuable evidence that aging-associated transcriptional programs are linked to patient outcome; however, comprehensive characterization of these signatures in relation to the immune microenvironment, tumor stemness, somatic mutation patterns, and cell-type-specific expression remains insufficient. Moreover, bulk transcriptomic analyses cannot resolve the cellular sources of candidate genes within the heterogeneous tumor ecosystem. Single-cell transcriptomics can help address this limitation [24, 25], while experimental validation is needed to support the biological relevance of computational findings.

In this study, we systematically identified aging-related differentially expressed genes in LUAD using TCGA and external GEO cohorts, defined aging-related molecular subtypes, and developed a prognostic signature for survival prediction. We further characterized its associations with clinicopathological features, immune infiltration, tumor stemness, and somatic mutation patterns, and established a nomogram for individualized prognostic evaluation. In addition, single-cell transcriptomic analysis, RT-qPCR validation in paired clinical samples, and preliminary in vitro experiments were performed to refine the biological interpretation of key genes. Collectively, this study provides an aging-related framework for prognostic assessment and offers biologically relevant insights into LUAD heterogeneity.

## Methods

### Data acquisition and preprocessing

Transcriptomic data and corresponding clinical information for LUAD were obtained from TCGA and the UCSC Xena browser [26, 27]. A total of 59 normal lung tissues and 528 LUAD tissues with complete survival data were included. TCGA RNA-seq data in FPKM format were converted into TPM values and transformed as log2(TPM + 1). Two GEO cohorts, GSE37745 and GSE50081, were used for external validation [28, 29]. For the GEO microarray cohorts, probes were annotated to official gene symbols using the corresponding platform files. Probes without valid gene symbols were excluded, and multiple probes mapping to the same gene were averaged. All eight signature genes were available in both GSE37745 and GSE50081. The TCGA-derived coefficients were applied to the processed GEO matrices to calculate risk scores. Given the RNA-seq/microarray platform differences, validation was performed using cohort-specific median risk scores for within-cohort stratification rather than absolute cross-platform calibration.

ARGs were retrieved from the GeneCards database using the keyword “aging” with a relevance score > 5 and from the HAGR database [30]. After removing duplicates, 2,543 ARGs were retained [31]. scRNA-seq data from GSE131907 were used to investigate the cellular expression patterns of key genes [32].

### Identification of aging-related DEGs and prognostic genes

Differential expression analysis between normal and LUAD tissues in the TCGA cohort was performed using the limma package [33]. Genes with *P* < 0.05 and |log2FC| > 1 were defined as DEGs. DEGs identified in TCGA were intersected with those from GSE37745 and GSE50081, and the overlapping genes were further intersected with ARGs to obtain aging-related DEGs.

Univariate Cox regression analysis was then performed using the survival package to identify prognosis-related aging genes. Genes with *P* < 0.01 were retained for subsequent analyses.

### Functional enrichment analysis

GO, KEGG, and DO enrichment analyses were performed using the clusterProfiler package [34]. Terms with *P* < 0.05 were considered significantly enriched.

### Consensus clustering analysis

Unsupervised consensus clustering was performed based on prognosis-related aging genes using the ConsensusClusterPlus package. The PAM algorithm with Euclidean distance was applied, with a resampling proportion of 0.8 and 1,000 iterations. The optimal number of clusters was determined according to the consensus matrix, CDF curve, and clustering stability. KM survival analysis and PCA were used to evaluate survival differences and sample distribution among subtypes.

### Immune infiltration and pathway analyses

Immune infiltration differences between molecular subtypes were evaluated using the xCell algorithm implemented in the immunedeconv package [35]. StromalScore, ImmuneScore, ESTIMATEScore, and tumor purity were calculated using the estimate package [36]. Pathway differences between subtypes were analyzed based on KEGG gene sets by GSVA.

### Construction and validation of the prognostic signature

Prognosis-related aging genes were further screened by LASSO-Cox regression using the glmnet package. The optimal penalty parameter was determined by ten-fold cross-validation [37]. A risk score was calculated for each patient according to the regression coefficients and gene expression levels. Patients in the TCGA cohort were classified into high-risk and low-risk groups using the median risk score as the cutoff. For external validation, the same eight-gene formula was applied to the GSE37745 and GSE50081 cohorts, and patients were stratified according to the median risk score within each external cohort.

To further evaluate model performance, bootstrap internal validation with 1,000 resampling iterations was performed in the TCGA cohort, and the bootstrap-corrected C-index was calculated. Calibration curves were generated to assess the agreement between predicted and observed survival probabilities. Decision curve analysis was performed to evaluate the potential clinical net benefit of the prognostic signature.

KM survival analysis and time-dependent ROC analysis were used to evaluate the predictive performance of the model in the TCGA cohort and the external GEO cohorts.

### Independent prognostic analysis and nomogram construction

Univariate and multivariate Cox regression analyses were performed to determine whether the risk score was an independent prognostic factor. A nomogram integrating the risk score and clinicopathological variables was constructed using the rms package to predict 1-, 3-, and 5-year OS. Calibration curves and ROC analyses were used to assess its predictive performance [38]. Collinearity among variables included in the nomogram was assessed using the degree-of-freedom-adjusted GVIF. The incremental value of the risk score was evaluated by comparing the C-index between the clinical model and the combined model incorporating the risk score.

### Association of the signature with clinicopathological features, somatic mutation, stemness, and the immune microenvironment

Differences in risk scores among clinicopathological subgroups were compared using the Wilcoxon rank-sum test. Somatic mutation data were obtained from UCSC Xena and analyzed using the maftools package [39, 40]. GSEA was performed to explore biological differences between the high-risk and low-risk groups. Correlation analysis between the risk score and RNAss was conducted to evaluate the association between the prognostic signature and tumor stemness. ssGSEA and ESTIMATE analysis were further used to characterize immune-related differences between risk groups.

### scRNA-seq analysis

scRNA-seq data from GSE131907 were used to evaluate the cell-type-specific expression pattern of CLDN14 in LUAD [32]. Five paired LUAD tumor and adjacent normal lung samples were included. Cells were analyzed using Seurat and retained if they had more than 200 detected genes and less than 15% mitochondrial gene content. After quality control, 29,634 genes and 37,952 cells were retained for downstream analysis.

Data were normalized using the LogNormalize method, and the top 2,000 highly variable genes were selected for PCA. Batch effects among samples were corrected using Harmony, and the top 20 Harmony dimensions were used for clustering and t-SNE visualization. Cell types were annotated using SingleR with multiple human reference datasets, and cluster marker genes were identified using FindAllMarkers to review the annotation results. CLDN14 expression was visualized using t-SNE feature plots and dot plots.

### Cell culture and transfection

The human LUAD cell lines A549 and H1299 were cultured in DMEM supplemented with 10% FBS in a humidified incubator at 37 °C with 5% CO2. siRNAs targeting CLDN14 and a negative control siRNA were used for transfection. Cells were transfected according to the manufacturer’s instructions and collected 24 h after transfection for subsequent experiments.

### RT-qPCR

Total RNA was extracted from paired tumor and adjacent normal tissues or cultured cells using TRIzol reagent and reverse-transcribed into cDNA. RT-qPCR was performed under the following conditions: 95 °C for 10 s, followed by 40 cycles of 95 °C for 10 s, 60 °C for 20 s, and 72 °C for 30 s. GAPDH was used as the internal control, and relative gene expression was calculated using the 2^-ΔΔCt method [41, 42].

The primer sequences were as follows:

- CLDN14 forward, 5′-CAGTAGCAAACCAAACCTCAGC-3′;
- CLDN14 reverse, 5′-TGGCGTCAATTTCATCAATAAAC-3′;
- GAPDH forward, 5′-GGAGCGAGATCCCTCCAAAAT-3′;
- GAPDH reverse, 5′-GGCTGTTGTCATACTTCTCATGG-3′.

### CCK-8 assay

Cell proliferation was assessed using a CCK-8 assay according to the manufacturer’s instructions. Transfected A549 and H1299 cells were seeded into plates at a density of 6 × 10^4 cells per well. At the indicated time points, CCK-8 reagent was added and absorbance at 450 nm was measured using a microplate reader.

### Transwell migration and invasion assays

Cell migration and invasion were assessed using Transwell chambers with 8-µm pore-size inserts. Transfected A549 cells were seeded into the upper chamber in serum-free DMEM, while medium containing 10% FBS was added to the lower chamber. For invasion assays, the upper chamber was precoated with Matrigel. After 48 h, cells on the lower surface were fixed, stained with crystal violet, and counted under an inverted microscope.

### Ethics approval and consent to participate

Paired tumor and adjacent normal tissues were collected from 10 LUAD patients at Renmin Hospital of Wuhan University. Total RNA was extracted and CLDN14 expression was measured by RT-qPCR. The study was conducted in accordance with the Declaration of Helsinki and was reviewed and approved by the Ethics Committee of Renmin Hospital of Wuhan University before the investigation. The ethical approval number was WDRY2024-K078 (dated April 9, 2024). The Ethics Committee granted a waiver of informed consent for this study.

### Statistical analysis

All statistical analyses were performed using R software (version 4.4.3). The random seed was set to 999,999 where applicable to improve reproducibility. Differences between two groups were assessed using the Wilcoxon rank-sum test or Student’s t test, as appropriate, and comparisons among more than two groups were performed using the Kruskal–Wallis test. Survival differences were evaluated using Kaplan–Meier analysis with the log-rank test, and correlations were assessed using Spearman’s correlation analysis. Paired clinical samples were analyzed using the paired Wilcoxon signed-rank test. For high-dimensional enrichment and immune-related analyses, P values were adjusted using the Benjamini–Hochberg method where appropriate. All tests were two-sided, and *P* < 0.05 or FDR < 0.05 was considered statistically significant.

## Results

### Identification and functional characterization of aging-related differentially expressed genes in LUAD

To characterize aging-related transcriptional alterations in lung adenocarcinoma (LUAD), we compared tumor and normal lung tissues in the TCGA cohort and identified 8,109 differentially expressed genes (DEGs). Overlap of these DEGs with those from the GEO validation cohorts GSE37745 and GSE50081 yielded 2,070 common DEGs (Supplementary Table S1). Intersecting these genes with the aging-related gene set (Supplementary Table S2) identified 274 aging-related DEGs (Supplementary Table S3).

**Fig. 1:**
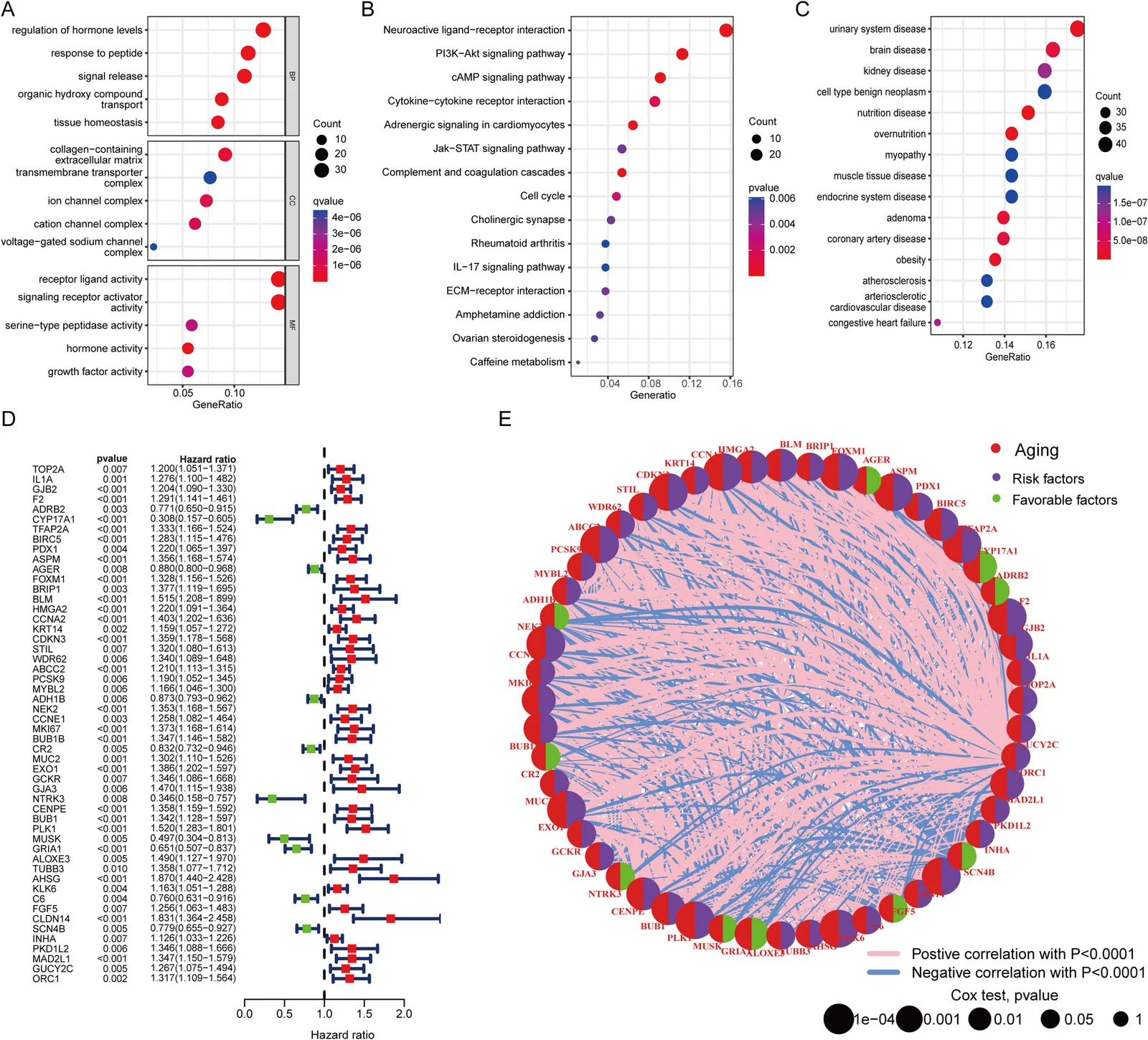
Identification and functional characterization of aging-related DEGs in LUAD. **A** GO enrichment analysis of 274 aging-related DEGs. **B** KEGG pathway enrichment analysis of aging-related DEGs. **C** DO enrichment analysis of aging-related DEGs. **D** Forest plot of univariate Cox regression analysis of prognosis-associated aging-related genes in LUAD. **E** Correlation network of prognosis-associated aging-related genes

To explore the potential functions of these genes, GO, KEGG, and DO enrichment analyses were performed. GO analysis indicated that these genes were mainly involved in regulation of hormone levels, tissue homeostasis, receptor ligand activity, and collagen-containing extracellular matrix (Fig. 1A). KEGG analysis showed significant enrichment in the PI3K–Akt signaling pathway, cytokine–cytokine receptor interaction, JAK–STAT signaling pathway, and IL-17 signaling pathway (Fig. 1B). DO analysis further revealed significant associations with endocrine system disease, obesity, atherosclerosis, and cardiovascular disease (Fig. 1C).

Univariate Cox regression analysis of the 274 aging-related DEGs identified 52 prognosis-associated genes with *P* < 0.01 (Fig. 1D). A gene–gene interaction network further demonstrated extensive correlations among these prognostic genes (Fig. 1E).

### Identification of aging-related molecular subtypes in LUAD

**Fig. 2:**
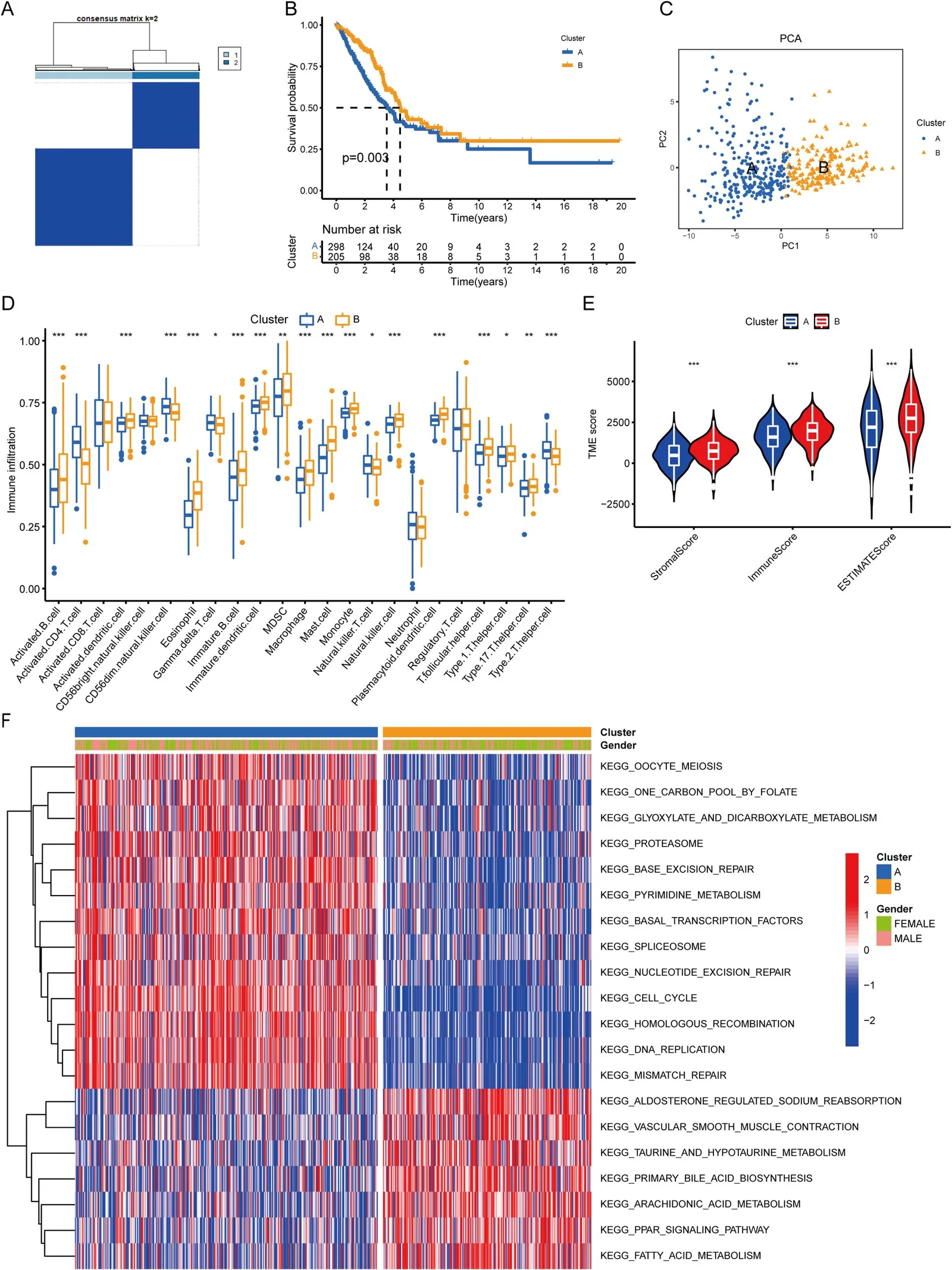
Identification of aging-related molecular subtypes in LUAD. **A** Consensus clustering matrix of LUAD samples based on 52 prognosis-associated aging-related genes at k = 2. **B** Kaplan–Meier analysis of OS between the two aging-related molecular subtypes. **C** PCA of the two aging-related molecular subtypes. **D** Comparison of immune cell infiltration between the two aging-related molecular subtypes. **E** Comparison of stromal score, immune score, and ESTIMATE score between the two aging-related molecular subtypes. **F** Heatmap of KEGG pathway enrichment in the two aging-related molecular subtypes. ns, not significant; *P < 0.05; **P < 0.01; ***P < 0.001

To investigate molecular heterogeneity in LUAD, unsupervised consensus clustering was performed based on the 52 aging-related prognostic genes. When k = 2, patients were stably classified into two molecular subtypes, designated cluster A and cluster B (Fig. 2A).

Kaplan–Meier analysis showed that patients in cluster B had significantly better overall survival than those in cluster A (*P* = 0.003; Fig. 2B). PCA further demonstrated a clear separation between the two subtypes (Fig. 2C).

Immune infiltration analysis revealed substantial differences in the tumor immune microenvironment between cluster A and cluster B (Fig. 2D). Several immune cell populations, including activated CD8 T cells, macrophages, and regulatory T cells, differed significantly between the two clusters. In addition, cluster B exhibited higher stromal, immune, and ESTIMATE scores than cluster A, indicating greater infiltration of immune and stromal components within the tumor microenvironment (Fig. 2E).

KEGG analysis further revealed distinct pathway enrichment patterns between the two subtypes. Cluster A was mainly enriched in cell cycle, DNA replication, and mismatch repair pathways, whereas cluster B was primarily enriched in PPAR signaling, fatty acid metabolism, and arachidonic acid metabolism (Fig. 2F).

### Development and validation of an aging-related prognostic signature in LUAD

**Fig. 3:**
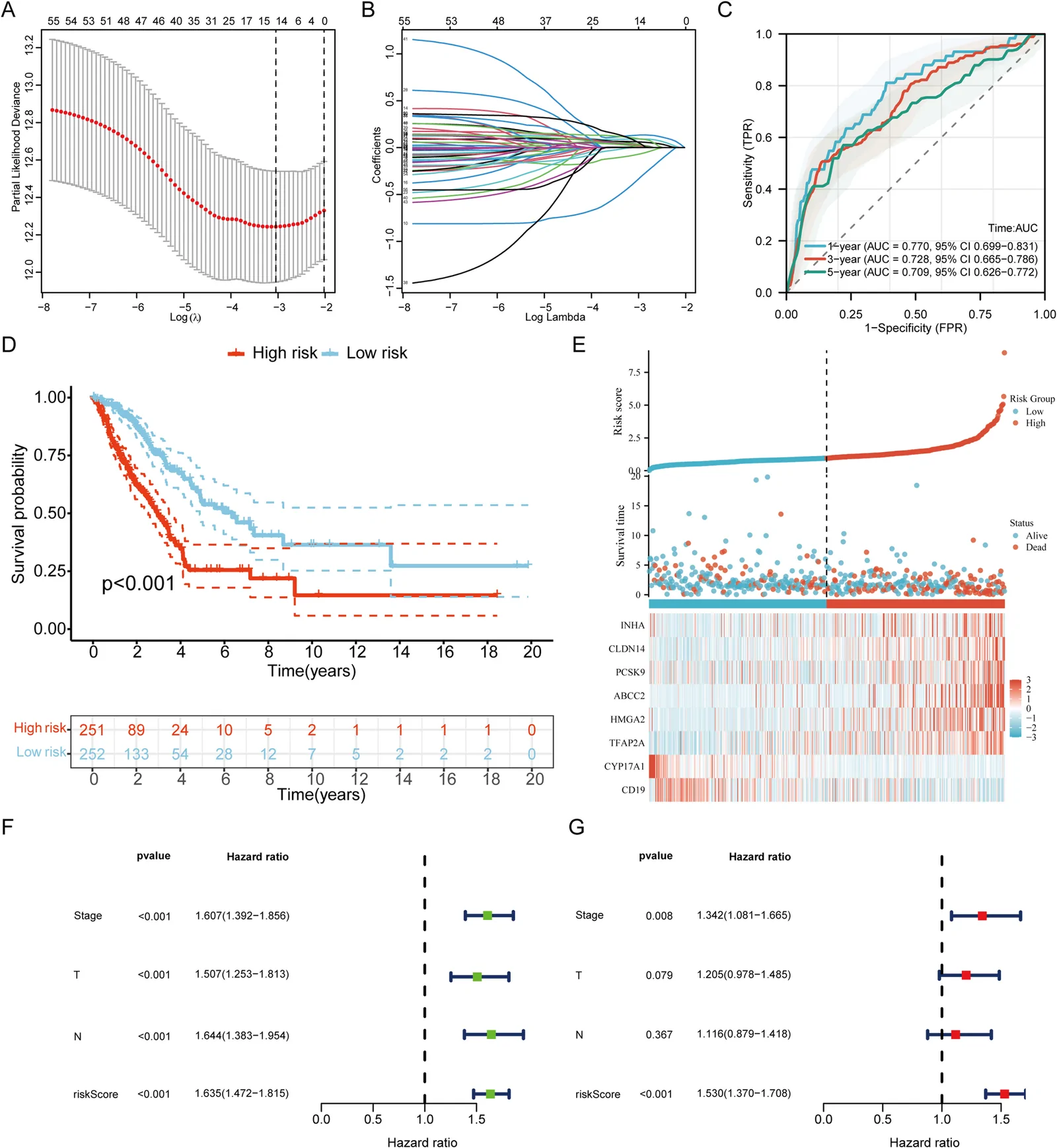
Development of an aging-related prognostic signature in LUAD. **A** Cross-validation plot for tuning parameter selection in the LASSO Cox regression analysis. **B** LASSO coefficient profiles of prognosis-associated aging-related genes. **C** Time-dependent ROC curves of the prognostic signature for predicting 1-, 3-, and 5-year OS. **D** Kaplan–Meier analysis of OS between the high-risk and low-risk groups. **E** Distribution of risk scores, survival status, and expression of the eight signature genes in the high-risk and low-risk groups. **F** Univariate Cox regression analysis of clinicopathological variables and the risk score in LUAD. **G** Multivariate Cox regression analysis of clinicopathological variables and the risk score in LUAD

To construct a prognostic model for LUAD, the 52 genes identified by univariate Cox regression were further subjected to LASSO-Cox regression analysis, and eight genes were ultimately selected to establish the prognostic signature: CD19, CYP17A1, TFAP2A, HMGA2, ABCC2, PCSK9, CLDN14, and INHA (Fig. 3A, B).

The risk score was calculated as follows:

$$\begin{aligned}\text{Risk score}\;=&-0.1977\;\times\;\mathrm{CD19}-1.0044\;\times\;\mathrm{CYP17A1}\\&+\;0.1103\times\;\mathrm{TFAP2A}+\;0.1735\;\times\;\mathrm{HMGA2}\\&+\;0.1167\;\times\;\mathrm{ABCC2}+\;0.1223\times\;\mathrm{PCSK9}\\&+\;0.4168\times\;\mathrm{CLDN14}\;+\;0.0900\;\times\;\mathrm{INHA}\end{aligned}$$

Patients were then stratified into high-risk and low-risk groups according to the median risk score. Time-dependent ROC analysis suggested moderate prognostic performance, with AUC values of 0.770 (95% CI: 0.699–0.831), 0.728 (95% CI: 0.665–0.786), and 0.709 (95% CI: 0.626–0.772) for 1-, 3-, and 5-year overall survival, respectively (Fig. 3C). Kaplan–Meier analysis showed that patients in the high-risk group had significantly worse overall survival than those in the low-risk group (*P* < 0.001; Fig. 3D).

As the risk score increased, survival status progressively worsened. Moreover, the eight signature genes exhibited distinct expression patterns between the two risk groups. Among them, CD19 and CYP17A1 had negative coefficients, whereas TFAP2A, HMGA2, ABCC2, PCSK9, CLDN14, and INHA had positive coefficients (Fig. 3E). Univariate and multivariate Cox regression analyses further confirmed that the risk score was an independent prognostic factor for overall survival (Fig. 3F, G).

To further evaluate the prognostic model, bootstrap internal validation was performed in the TCGA cohort. The bootstrap-corrected C-index was 0.703 based on 1,000 resampling iterations, indicating moderate discriminative ability (Supplementary Table S4). Calibration curves showed acceptable agreement between predicted and observed survival probabilities (Supplementary Figure S1A-C), and decision curve analysis suggested potential clinical net benefit within a certain range of threshold probabilities (Supplementary Figure S1D-F).

**Fig. 4:**
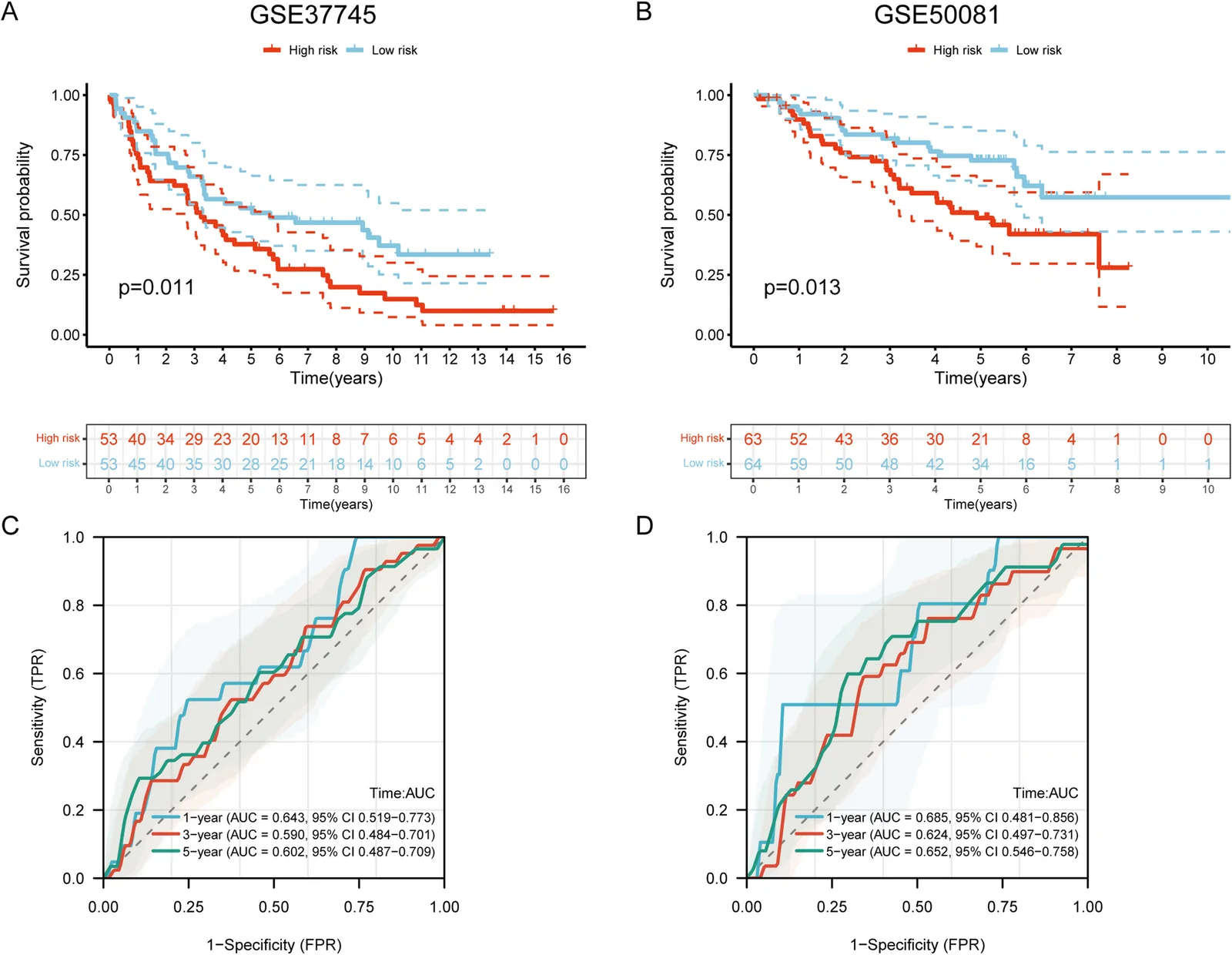
External validation of the aging-related prognostic signature in GEO cohorts. **A** Kaplan–Meier analysis of OS in the GSE37745 cohort. **B** Kaplan–Meier analysis of OS in the GSE50081 cohort. **C** Time-dependent ROC curves in the GSE37745 cohort. **D** Time-dependent ROC curves in the GSE50081 cohort

To further validate the prognostic value of the signature, two independent GEO cohorts, GSE37745 and GSE50081, were used for external validation. After probe-to-gene mapping and expression preprocessing, all eight model genes were available in both GEO cohorts. Patients in both cohorts were assigned risk scores using the same TCGA-derived formula and stratified into high- and low-risk groups according to the median risk score within each cohort. Kaplan–Meier analysis showed that patients in the high-risk group had significantly worse overall survival than those in the low-risk group in both GSE37745 (*P* = 0.011; Fig. 4A) and GSE50081 (*P* = 0.013; Fig. 4B). Time-dependent ROC analysis yielded AUC values of 0.643 (95% CI: 0.519–0.773), 0.590 (95% CI: 0.484–0.701), and 0.602 (95% CI: 0.487–0.709) for 1-, 3-, and 5-year overall survival in GSE37745, respectively (Fig. 4C). In GSE50081, the corresponding AUC values were 0.685 (95% CI: 0.481–0.856), 0.624 (95% CI: 0.497–0.731), and 0.652 (95% CI: 0.546–0.758), respectively (Fig. 4D).

### Clinical relevance of the risk score and nomogram construction

**Fig. 5:**
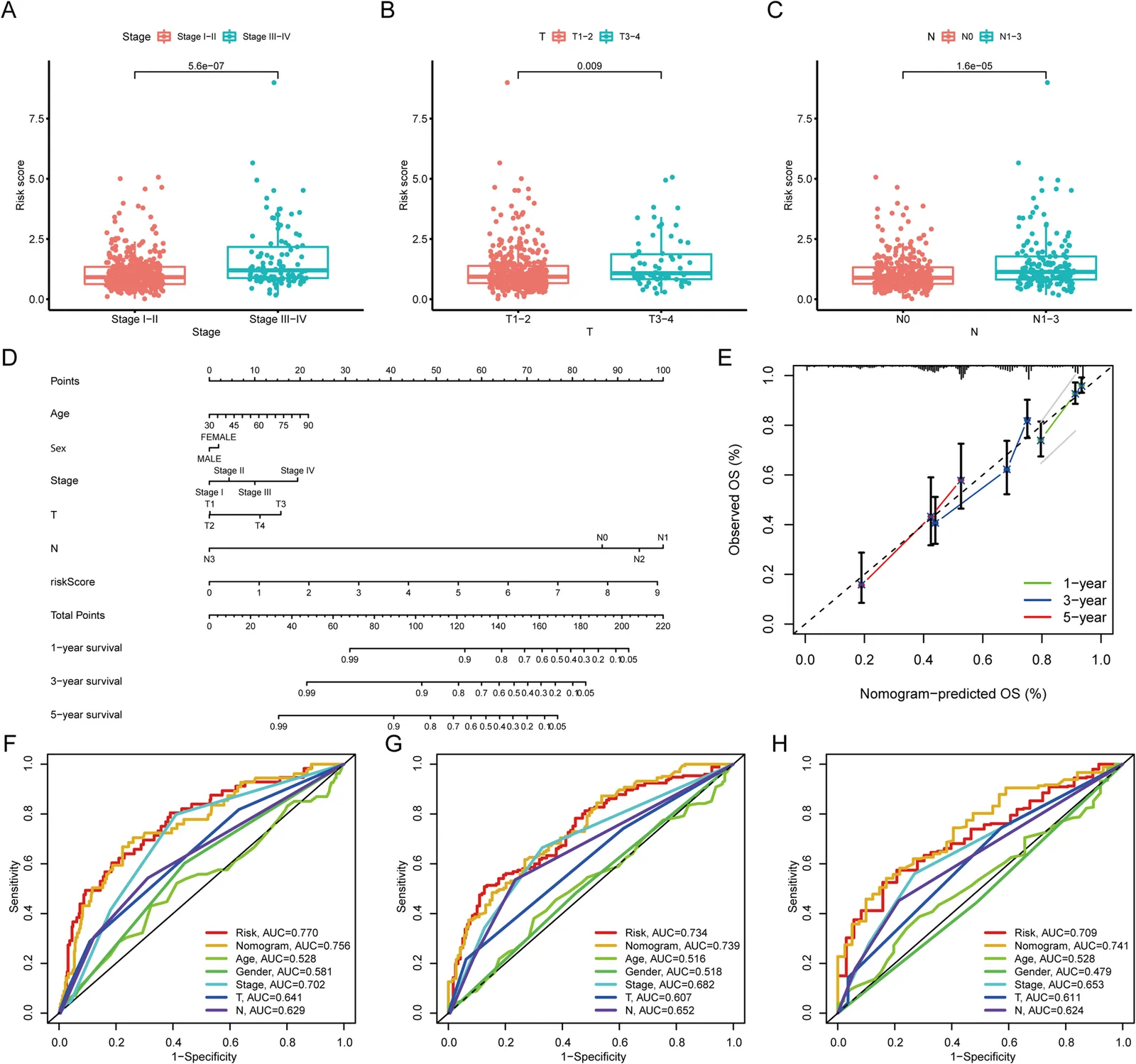
Clinical relevance of the risk score and nomogram construction. **A** Comparison of risk scores between stage I–II and stage III–IV patients. **B** Comparison of risk scores between T1–2 and T3–4 patients. **C** Comparison of risk scores between N0 and N1–3 patients. **D** Nomogram for predicting 1-, 3-, and 5-year OS in LUAD. **E** Calibration curves of the nomogram for 1-, 3-, and 5-year OS. **F**–**H** Time-dependent ROC curves comparing the predictive performance of the nomogram, risk score, and clinicopathological variables for 1-year (**F**), 3-year (**G**), and 5-year (**H**) OS

We next evaluated the distribution of risk scores across clinicopathological subgroups. Risk scores were significantly higher in patients with stage III–IV disease than in those with stage I–II disease (*P* < 0.001; Fig. 5A). Similarly, patients with T3–4 tumors had higher risk scores than those with T1–2 tumors (*P* = 0.009; Fig. 5B), and patients with N1–3 disease had higher risk scores than those with N0 disease (*P* < 0.001; Fig. 5C).

To improve individualized prognostic prediction, a nomogram integrating age, sex, stage, T stage, N stage, and risk score was constructed (Fig. 5D). Calibration curves for 1-, 3-, and 5-year overall survival showed acceptable agreement between predicted and observed survival probabilities (Fig. 5E). GVIF analysis did not indicate severe multicollinearity among variables included in the nomogram (Supplementary Table S5).

The predictive performance of the nomogram was further compared with that of conventional clinicopathological variables and the risk score alone. The nomogram achieved AUC values of 0.756, 0.739, and 0.741 for 1-, 3-, and 5-year overall survival, respectively, which were generally superior to those of most conventional clinicopathological variables (Fig. 5F–H). Compared with the clinical model alone, the combined clinical-risk model achieved a higher C-index (0.721 vs. 0.669; Supplementary Table S6), suggesting incremental prognostic value of the risk score.

### Somatic mutation patterns and functional characteristics of the risk subgroups

**Fig. 6:**
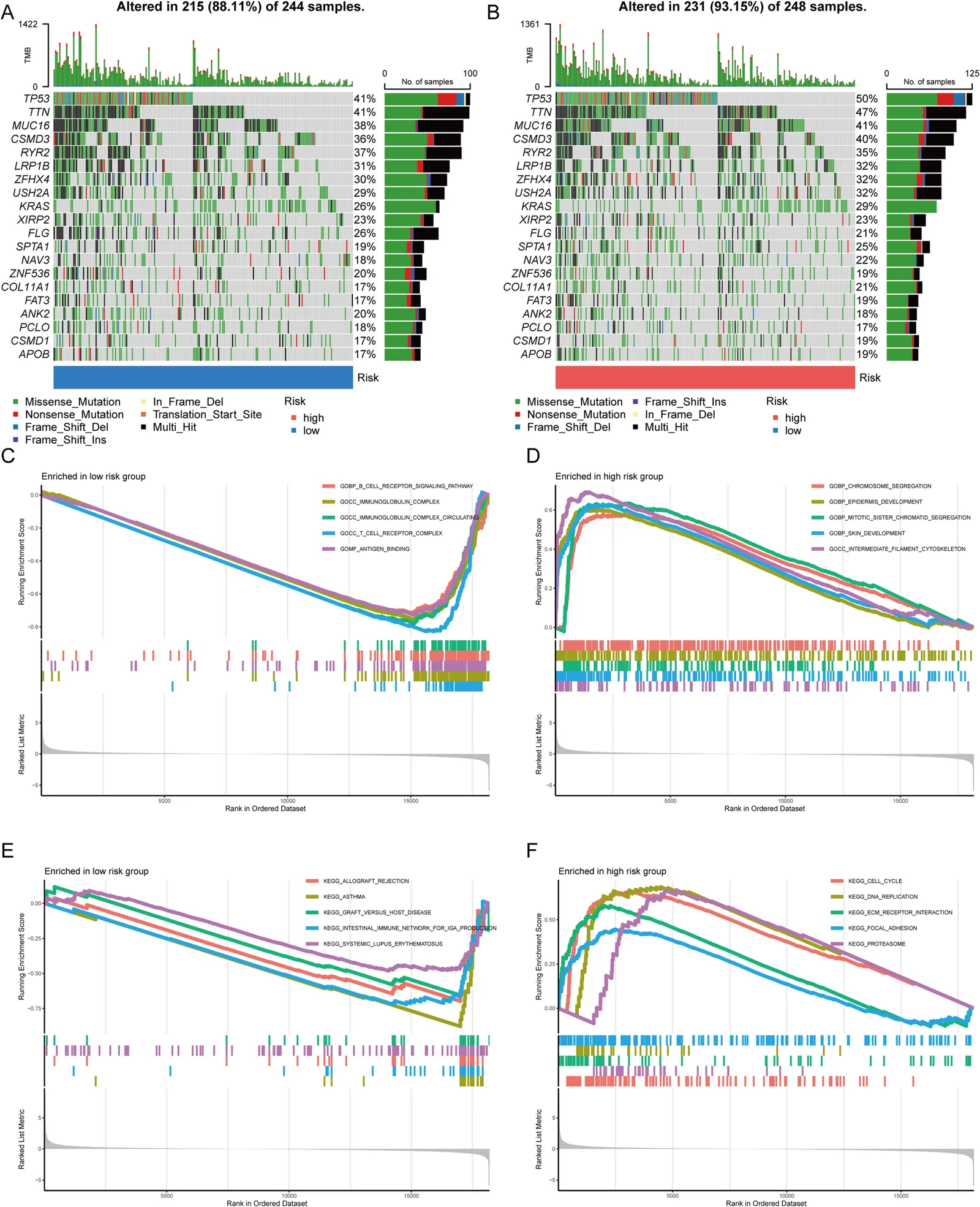
Somatic mutation patterns and functional characteristics of the risk subgroups. **A**, **B** Somatic mutation landscapes of the low-risk group (**A**) and high-risk group (**B**). **C**, **D** GO-based GSEA of pathways enriched in the low-risk group (**C**) and high-risk group (**D**). **E**, **F** KEGG-based GSEA of pathways enriched in the low-risk group (**E**) and high-risk group (**F**)

Somatic mutation analysis revealed distinct genomic features between the two risk groups. The high-risk group exhibited a higher somatic mutation frequency than the low-risk group, with mutations detected in 231 of 248 samples (93.15%) versus 215 of 244 samples (88.11%), respectively (Fig. 6A, B).

To further characterize the biological differences between the two groups, GSEA was performed. In the low-risk group, GO terms were mainly enriched in immune-related processes, including B-cell receptor signaling and T-cell receptor complex (Fig. 6C). KEGG analysis likewise showed enrichment of immune-associated pathways, such as allograft rejection and the intestinal immune network for IgA production (Fig. 6E).

In contrast, the high-risk group was mainly enriched in pathways associated with proliferation and tumor progression. GO analysis highlighted chromosome segregation and mitotic sister chromatid segregation (Fig. 6D), whereas KEGG analysis showed enrichment in cell cycle, DNA replication, and ECM–receptor interaction pathways (Fig. 6F).

### Associations of the prognostic signature with stemness and the immune microenvironment

**Fig. 7:**
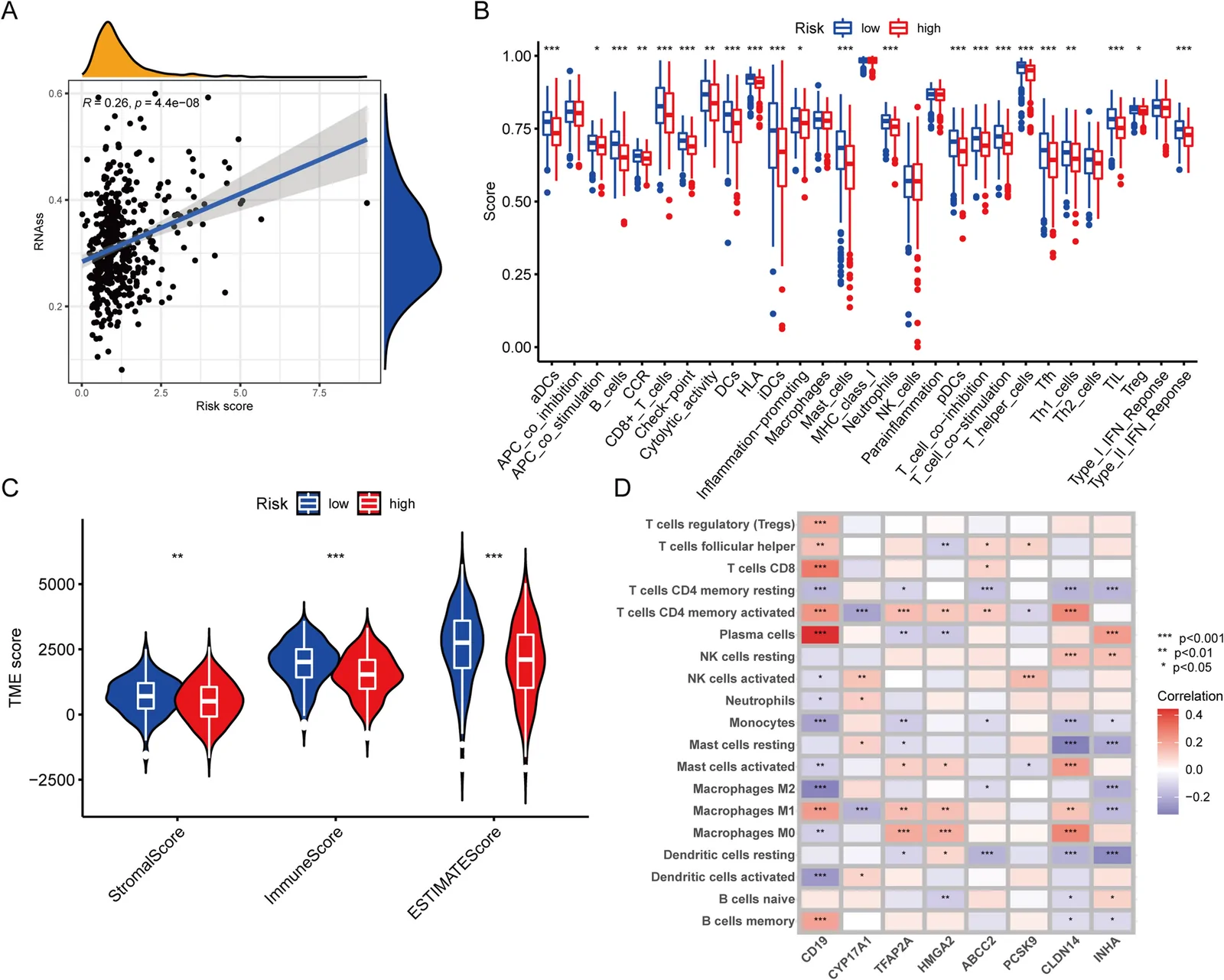
Association of the prognostic signature with stemness and the immune microenvironment. **A** Correlation between the risk score and RNAss. **B** Comparison of immune-related signatures between the low-risk and high-risk groups based on ssGSEA. **C** Comparison of stromal score, immune score, and ESTIMATE score between the low-risk and high-risk groups. **D** Correlation between the eight signature genes and immune-infiltrating cells. ns, not significant; *P < 0.05; **P < 0.01; ***P < 0.001

We next assessed the associations of the prognostic signature with tumor stemness and the immune microenvironment. Correlation analysis showed that the risk score was positively correlated with RNAss (*R* = 0.26, *P* < 0.001; Fig. 7A).

ssGSEA of immune-related functions revealed significant differences in multiple immune signatures between the two risk groups, with generally higher enrichment scores in the low-risk group (Fig. 7B). Consistently, ESTIMATE analysis showed that StromalScore, ImmuneScore, and ESTIMATEScore were significantly higher in the low-risk group (Fig. 7C).

Correlation analysis between the signature genes and immune-infiltrating cells further showed that the eight model genes were differentially associated with multiple immune cell subsets (Fig. 7D).

### Single-cell expression pattern and preliminary functional assessment of CLDN14 in LUAD

**Fig. 8:**
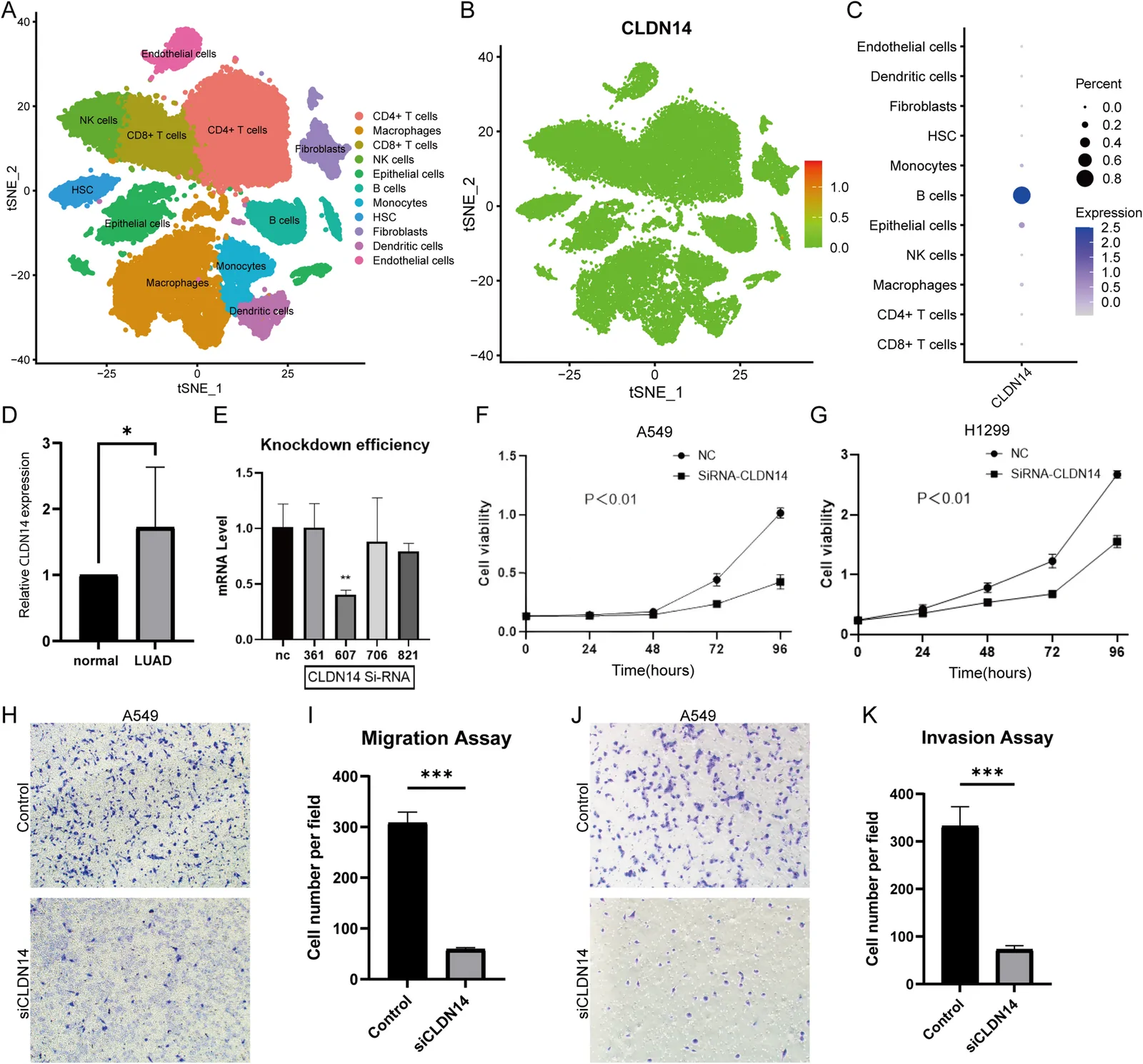
Single-cell expression pattern and functional validation of CLDN14 in LUAD. **A** t-SNE plot showing the major cell clusters identified in the scRNA-seq dataset. **B** t-SNE feature plot showing the distribution of CLDN14 expression in the scRNA-seq dataset. **C** Dot plot showing CLDN14 expression across different cell types. **D** RT-qPCR analysis of CLDN14 expression in paired LUAD tissues and matched adjacent normal tissues, (*n* = 10 pairs). **E** Knockdown efficiency of four siRNAs targeting CLDN14. **F**, **G** CCK-8 assays showing the effects of CLDN14 knockdown on cell viability in A549 (**F**) and H1299 (**G**) cells. **H**, **I** Representative images (**H**) and quantification (**I**) of the Transwell migration assay in A549 cells after CLDN14 knockdown. **J**, **K** Representative images (**J**) and quantification (**K**) of the Transwell invasion assay in A549 cells after CLDN14 knockdown. For in vitro assays, experiments were independently repeated three times, and data are presented as mean ± SD. Paired tissue comparisons were performed using the paired Wilcoxon signed-rank test. In vitro comparisons were performed using Student’s t test or one-way ANOVA, as appropriate. ns, not significant; *P < 0.05; **P < 0.01; ***P < 0.001

Single-cell RNA-seq analysis identified multiple immune and stromal cell populations in LUAD. Among these cell types, CLDN14 showed relatively higher expression in B cells than in other annotated cell populations (Fig. 8A–C). In addition, we evaluated CLDN14 expression in paired tumor and adjacent normal samples from 10 LUAD patients. RT-qPCR results showed that CLDN14 expression was significantly higher in tumor tissues than in adjacent normal tissues (*P* = 0.027; Fig. 8D).

RT-qPCR was performed to evaluate the knockdown efficiency of four siRNAs targeting CLDN14. Among them, siRNA-607 showed the highest silencing efficiency, reducing CLDN14 mRNA expression to approximately 40% of that in the control group (*P* < 0.01; Fig. 8E).

The effect of CLDN14 knockdown on LUAD cell proliferation was subsequently assessed using the CCK-8 assay. Compared with the negative control group, CLDN14 silencing significantly inhibited the proliferation of both A549 and H1299 cells (*P* < 0.01; Fig. 8F, G).

To further determine the role of CLDN14 in LUAD cell motility, Transwell migration and invasion assays were performed in A549 cells. Representative images showed that the numbers of migrated and invaded cells were markedly reduced in the siCLDN14 group compared with the control group (Fig. 8H, J). Consistently, quantitative analysis demonstrated that CLDN14 knockdown significantly suppressed both migratory and invasive capacities of A549 cells (*P* < 0.001; Fig. 8I, K).

## Discussion

In this study, we systematically characterized aging-related molecular alterations in LUAD and developed an eight-gene prognostic signature that stratified patients according to overall survival. By integrating bulk transcriptomic profiling with clinicopathological features, immune infiltration, stemness characteristics, somatic mutation patterns, single-cell analysis, and preliminary in vitro validation, we established an aging-related framework for prognostic assessment in LUAD. In addition to showing prognostic relevance in independent cohorts, this signature may provide preliminary insight into the biological heterogeneity of LUAD.

Our findings further suggest that aging-related transcriptional programs are closely involved in LUAD progression and phenotypic diversity. Rather than merely reflecting chronological aging, the prognostic relevance of this signature may lie in its ability to capture coordinated biological states associated with both tumor-intrinsic aggressiveness and microenvironmental remodeling. This broader biological relevance may help explain why aging-related genes provide meaningful prognostic information in LUAD [11, 16, 43].

Consistent with this interpretation, the two risk groups defined by our model exhibited distinct biological and clinical characteristics. High-risk tumors were associated with worse survival, more advanced clinicopathological features, higher stemness indices, and enrichment of proliferation-related pathways. By contrast, low-risk tumors showed higher immune- and stromal-related scores, suggesting greater immune and stromal components within the tumor microenvironment. These findings indicate that the prognostic performance of the signature may reflect the combined influence of tumor-intrinsic proliferative activity, stemness-associated traits, and microenvironmental composition. However, these transcriptome-based immune features should be interpreted cautiously and do not necessarily indicate enhanced antitumor immune activity.

The eight genes included in the signature represent distinct but biologically relevant aspects of aging-related tumor progression, involving immune remodeling, transcriptional regulation, metabolic reprogramming, epithelial plasticity, and treatment resistance. CD19, a canonical B-cell marker, exerted a protective effect in the model, suggesting that its contribution may reflect B-cell infiltration or humoral immune activity within the tumor microenvironment rather than a tumor cell-intrinsic oncogenic function [44]. In contrast, ABCC2, an ATP-binding cassette transporter involved in drug efflux, carried a positive coefficient and may indicate treatment resistance and a more aggressive tumor phenotype [45]. PCSK9 is a lipid metabolism-related gene that regulates cholesterol homeostasis and has also been implicated in tumor progression and immune regulation, suggesting a possible link between aging-associated metabolic remodeling and tumor immunity [46]. TFAP2A has been associated with LUAD progression and metastasis, including regulation through a miR-16 family/TFAP2A/PSG9/TGF-β signaling axis [47, 48], while HMGA2 is a chromatin-associated regulator involved in lung cancer proliferation and metastasis [49]. INHA, a component of the inhibin/activin signaling system, may be related to tumor progression and immune-suppressive tumor microenvironment remodeling in LUAD [50]. CYP17A1 is involved in steroid hormone biosynthesis and may represent endocrine- or metabolism-related alterations in LUAD, although its direct mechanistic role in this disease remains insufficiently defined [51]. Although the precise roles of several signature genes in LUAD remain incompletely understood, their combined prognostic value suggests convergence on biologically meaningful features of aging-related tumor progression.

Among the eight signature genes, CLDN14 was selected for further exploratory analysis because of its relatively large positive coefficient in the prognostic model and its potential biological relevance. Single-cell RNA-seq analysis showed that CLDN14 had the highest average expression in B cells among the annotated cell populations, suggesting that CLDN14 expression in bulk LUAD tissues may partly reflect immune microenvironmental components. Biologically, this finding may indicate an association between CLDN14 expression and tumor-infiltrating B cells or B-cell-enriched immune components within the LUAD microenvironment. In addition, given that CLDN14 is a tight junction-associated molecule, its expression pattern may also reflect potential interactions between malignant epithelial cells and immune microenvironmental components. Therefore, CLDN14 should not be interpreted simply as a tumor-cell-specific marker based on bulk transcriptomic data. Consistent with this caution, RT-qPCR analysis of paired clinical samples showed higher CLDN14 expression in LUAD tissues than in adjacent normal tissues, but this bulk-tissue result cannot determine whether the increased CLDN14 signal originated from tumor epithelial cells, B cells, or other stromal and immune populations.

In contrast, the in vitro knockdown assays were designed to evaluate whether CLDN14 has functional relevance when expressed in LUAD cell models. CLDN14 silencing reduced cell proliferation in A549 and H1299 cells and suppressed migration and invasion in A549 cells, suggesting a possible cell-intrinsic effect in these models. However, the B-cell-enriched expression pattern observed in single-cell analysis and the functional effects observed in LUAD cell lines represent different biological contexts. Thus, CLDN14 may have context-dependent relevance in LUAD, but this possibility remains speculative and requires further validation. Therefore, we interpret CLDN14 as a candidate gene of interest rather than a fully established mechanistic driver of LUAD progression.

CLDN14 belongs to the claudin family and encodes a tight junction-associated transmembrane protein involved in epithelial barrier integrity and paracellular permeability [52]. Increasing evidence suggests that aberrant CLDN14 expression participates in the development and progression of multiple malignancies, although its biological role appears to be highly tumor type-dependent [53, 54]. For example, CLDN14 has been reported to be epigenetically silenced by EZH2-mediated H3K27me3 in hepatocellular carcinoma and to be associated with patient prognosis [55]. In colorectal cancer, CLDN14 has been implicated in tumor progression and chemoresistance [56, 57], whereas in breast cancer it has also shown potential prognostic relevance [58]. These observations support the view that CLDN14 functions as a context-dependent regulator whose biological significance varies according to tissue origin and tumor microenvironmental context.

In lung cancer, however, direct evidence regarding CLDN14 remains limited. A previous study showed that circKIF4A promotes the proliferation and metastasis of non-small cell lung cancer cells through the miR-1238/CLDN14 axis, suggesting that CLDN14 may participate in lung cancer progression [59]. Consistent with this possibility, our results support an association between CLDN14 and malignant phenotypes in LUAD. Given its known role in tight junction biology, the effects of CLDN14 in LUAD may be related to altered tight junction dynamics, cell–cell adhesion, epithelial plasticity, and tumor-associated signaling processes. Previous studies in other tumor contexts have shown that metastasis-related processes, including MMP-mediated extracellular matrix remodeling, TGF-β-related epithelial plasticity, invasive metastatic structures, and tumor–microenvironment interactions, may contribute to metastatic progression [60, 61]. Therefore, whether CLDN14 influences LUAD progression through similar metastasis-related mechanisms remains an important question for future investigation. Nevertheless, because direct mechanistic evidence in LUAD remains limited, the precise role of CLDN14 and the molecular basis underlying its effects require further investigation through more comprehensive in vitro and in vivo studies.

From a clinical perspective, our study provides an aging-related prognostic model that may complement conventional stage-based assessment rather than replace it. The nomogram integrating the risk score with clinicopathological variables showed acceptable predictive performance, indicating its potential utility for individualized prognostic evaluation. More importantly, our findings suggest that aging-related transcriptional patterns may be informative not only for survival prediction but also for understanding biological heterogeneity in LUAD. In this regard, such models may help identify patient subgroups with distinct tumor states and potentially different therapeutic vulnerabilities.

Several limitations of this study should be acknowledged. First, this was a retrospective study based primarily on public transcriptomic datasets. Although external validation cohorts were included, prospective clinical validation is still required to confirm the robustness and clinical applicability of the aging-related prognostic signature. Second, the RT-qPCR validation was performed using a relatively limited number of paired clinical samples, and larger independent cohorts are needed to further verify the expression patterns and prognostic relevance of the signature genes. Third, although single-cell analysis improved the biological interpretability of the findings, the prognostic model was developed from bulk transcriptomic data and therefore could not fully resolve the cellular origin of gene-expression signals. Finally, the experimental validation focused mainly on CLDN14 and should be regarded as preliminary. Further protein-level validation, rescue experiments, in vivo studies, and mechanistic investigations are needed to clarify the functional roles of CLDN14 and other signature genes in LUAD.

## Conclusion

This study systematically characterized aging-related molecular alterations in LUAD, delineating their dysregulated expression patterns, molecular subtypes, prognostic relevance, and associations with immune-related features. Using eight aging-related genes, we constructed a prognostic signature that was able to stratify patients by overall survival in the TCGA cohort and showed prognostic relevance in two external validation cohorts, with associations with distinct stemness, somatic mutation, and tumor microenvironment characteristics. In addition, single-cell analysis, RT-qPCR validation in paired clinical samples, and in vitro experiments highlighted CLDN14 as a candidate gene potentially associated with LUAD progression. Together, these findings provide an initial aging-related framework for prognostic stratification and biological characterization in LUAD.

## Supplementary Information

Supplementary Material 1: Supplementary Table S1 presents the common differentially expressed genes obtained by overlapping DEGs from the TCGA-LUAD cohort with those from the GEO validation cohorts GSE37745 and GSE50081. Supplementary Table S2 contains the aging-related gene set used in this study. Supplementary Table S3 lists the aging-related differentially expressed genes identified by intersecting the common DEGs with the aging-related gene set. Supplementary Table S4 presents the bootstrap internal validation results of the eight-gene prognostic signature. Supplementary Table S5 presents the GVIF analysis of variables included in the nomogram. Supplementary Table S6 presents the C-index comparison between the clinical model and the combined clinical-risk model.

Supplementary Material 2: Figure S1 shows the calibration curves and decision curve analysis of the eight-gene prognostic signature in the TCGA cohort.

## Data Availability

Data Availability Statement: The datasets analyzed in this study are publicly available. Transcriptomic and clinical data for lung adenocarcinoma were obtained from TCGA and the UCSC Xena browser, and external validation datasets were obtained from GEO under accession numbers GSE37745, GSE50081, and GSE131907. The experimental data generated in this study, including RT-qPCR and in vitro assay data, are available from the corresponding author upon reasonable request.
